# Rapid *IDH1*-R132 genotyping panel utilizing locked nucleic acid loop-mediated isothermal amplification

**DOI:** 10.1093/biomethods/bpae012

**Published:** 2024-02-21

**Authors:** Kristian A Choate, Edward J Raack, Paul B Mann, Evan A Jones, Robert J Winn, Matthew J Jennings

**Affiliations:** Department of Biology, Northern Michigan University, Marquette, MI, United States; Upper Michigan Brain Tumor Center, Marquette, MI, United States; Upper Michigan Brain Tumor Center, Marquette, MI, United States; School of Clinical Sciences, Northern Michigan University, Marquette, MI, United States; Upper Michigan Brain Tumor Center, Marquette, MI, United States; School of Clinical Sciences, Northern Michigan University, Marquette, MI, United States; Applied Research Lab for Intelligence and Security, College Park, MD, United States; Department of Biology, Northern Michigan University, Marquette, MI, United States; Upper Michigan Brain Tumor Center, Marquette, MI, United States; Upper Michigan Brain Tumor Center, Marquette, MI, United States; School of Clinical Sciences, Northern Michigan University, Marquette, MI, United States

**Keywords:** IDH (Isocitrate dehydrogenase), Locked Nucleic Acids (LNA), Loop Mediated Isothermal Amplification (LAMP), genotyping panel, SNV (single nucleotide variants), glioma, mutant IDH1 glioma, IDH1 R132H, SNV discrimination

## Abstract

While the detection of single-nucleotide variants (SNVs) is important for evaluating human health and disease, most genotyping methods require a nucleic acid extraction step and lengthy analytical times. Here, we present a protocol which utilizes the integration of locked nucleic acids (LNAs) into self-annealing loop primers for the allelic discrimination of five isocitrate dehydrogenase 1 R132 (*IDH1*-R132) variants using loop-mediated isothermal amplification (LAMP). This genotyping panel was initially evaluated using purified synthetic DNA to show proof of specific SNV discrimination. Additional evaluation using glioma tumor lysates with known *IDH1*-R132 mutational status demonstrated specificity in approximately 35 min without the need for a nucleic acid extraction purification step. This LNA-LAMP-based genotyping assay can detect single base differences in purified nucleic acids or tissue homogenates, including instances where the variant of interest is present in an excess of background wild-type DNA. The pH-based colorimetric indicator of LNA-LAMP facilitates convenient visual interpretation of reactions, and we demonstrate successful translation to an end-point format using absorbance ratio, allowing for an alternative and objective approach for differentiating between positive and negative reactions. Importantly, the LNA-LAMP genotyping panel is highly reproducible, with no false-positive or false-negative results observed.

## Introduction

Historically, the development of convenient methods for detecting single-nucleotide variants (SNVs) has been challenging but remains important in the diagnosis of inherited diseases, the identification of somatic mutations associated with cancer, and the characterization of pharmacogenetic variants. Currently, sequencing, microarrays, or allele-specific PCR are commonly used for detecting SNVs but are time-consuming and require preanalytical nucleic acid purification; thus, these techniques often require hours to days to provide results. Loop-mediated isothermal amplification (LAMP) is a convenient nucleic acid analysis method due to its high sensitivity, specificity, and ability to utilize crude lysates. In previous work, we have described a colorimetric peptide nucleic acid LAMP (CPNA-LAMP) assay capable of specifically recognizing a missense mutation within the isocitrate dehydrogenase 1 (*IDH1*) gene in under an hour without the need for a nucleic acid extraction step [1]. Here, we expand upon this work by employing locked nucleic acids (LNAs) to mediate SNV discrimination. This allowed for the creation of a genotyping panel that targets the five most frequently encountered *IDH1*-R132 SNVs [2] (Table 1).

**Table 1.** Frequency and codon sequences of *IDH1*-R132 variants in low-grade glioma

*IDH1* R132 variant	Codon sequence	Frequency in low-grade glioma (%)
R132H	C**A**T	62–93
R132C	**T**GT	2.9–4.3
R132G	**G**GT	1.0–2.5
R132S	**A**GT	1.1–2.2
R132L	C**T**T	0.2–0.6

Bolded nucleotides are representative of each variant’s respective single nucleotide substitution.

**Figure 1.**
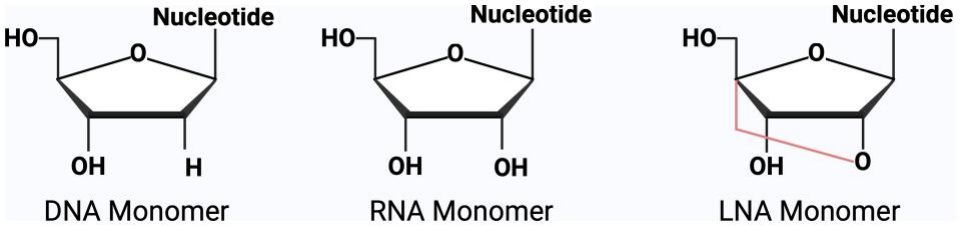
Structural differences between DNA, RNA, and LNA monomers. LNAs contain a methylene bridge that connects the 2′-oxygen with the 4′-carbon of ribose, resulting in a locked 3′-endo conformation that reduces conformational flexibility.

LNAs are RNA derivatives that have improved binding affinity to complementary bases in comparison to conventional oligonucleotides. LNA monomers most closely resemble RNA monomers but contain a methylene bridge between the 2′-oxygen and 4′- carbon, thereby constraining flexibility [3] (Fig. 1). LNA–DNA duplexes have superior stability due to a more favorable thermodynamic state; thus, the melting temperature of an LNA–DNA hybrid is higher than that of a corresponding DNA–DNA hybrid [4]. High stability, adherence to Watson–Crick–Franklin base-pairing rules, and mismatch discrimination [5] make the LNA a favorable addition to LAMP primers [6].

**Figure 2.**
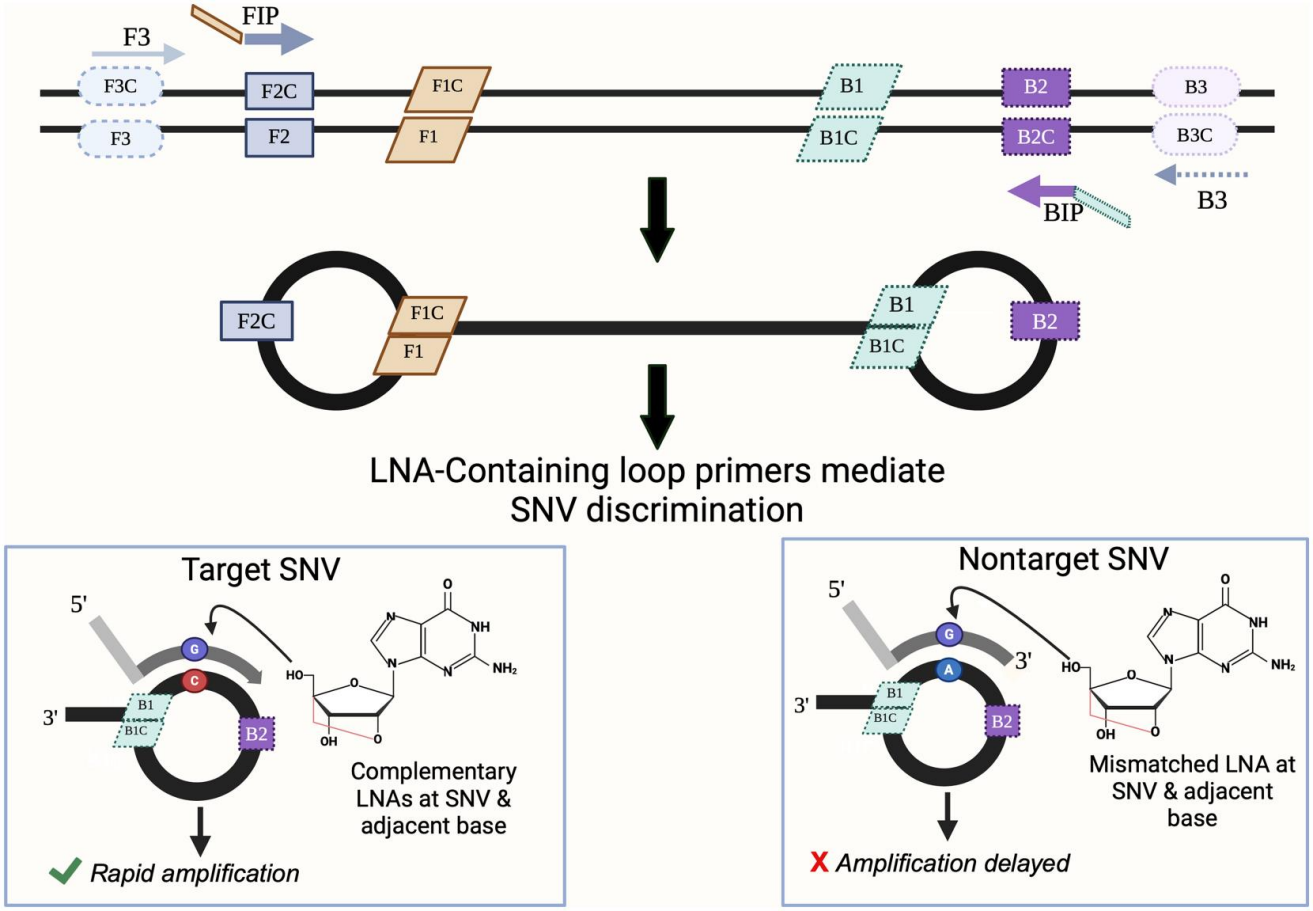
LNA-LAMP employs a biochemically modified SALP containing LNAs within the loop structure. Conventional LAMP employs four primers recognizing six distinct sequences. Strand displacement by the outer primers generate dumbbell structures which are targets for exponential amplification. In the design shown here, the LNA-SALPs target the backward loop, where *IDH1*-R132 SNVs are located. The four bases at the 5′-end of the SALP are not native to *IDH1*-R132 but were incorporated to create the hairpin structure in its unbound state. This figure was modified from Itonaga *et al*. [7].

Previously, we developed a peptide nucleic acid (PNA)-LAMP assay that uses conventional primers to amplify the region of the *IDH1* gene containing the R132 SNV site. This method relies upon an *IDH1*-R132 wild-type-specific PNA clamping probe combined with a self-annealing loop primer (SALP) that targets the R132H variant to impart single base specificity. Here, we modified this assay by incorporating LNAs into SALPs, eliminating the need for a clamping PNA. Additionally, this reduced reaction times and improved the ability to discriminate between SNVs. While the integration of an LNA into a SALP has been previously demonstrated to lower the limit of detection (LOD) and heighten specificity [7], the use of LAMP for SNV recognition relying solely upon the incorporation of LNAs without the presence of PNAs has not yet been demonstrated. In this study, the strategic addition of LNAs into SALPs that target various bases within the R132 codon permits the specific detection of multiple *IDH1*-R132 SNVs (Fig. 2).

In this study, we describe the generation of the first LNA-LAMP genotyping panel to discriminate between multiple different single base mutations in the same codon. Through optimization of this panel, we found the number and position of the LNAs in the SALP that target the codon bearing the SNV to be highly significant. Though the initial optimization was performed via colorimetric LNA-LAMP with a visual endpoint analysis, we also demonstrated the ability to shift the assay to an endpoint format by determining the absorbance ratio (430 nm/560 nm). Absorbance ratio analysis allows for a qualitative genotyping method and eliminates the subjectivity of colorimetric interpretation. Importantly, this method is rapid, accurate, and can simultaneously detect the five most common *IDH1*-R132 SNVs in approximately 35 min.

## Materials and methods

### Primer design

LAMP primers were created using Eiken’s PrimerExplorer primer design tool V4 (https://primerexplorer.jp/e/). Oligonucleotides were obtained from Integrated DNA Technologies (IDTs). During optimization, various quantities and positions of LNAs were tested within self-annealing loop primers for specificity to the respective variant (Table 2).

**Table 2.** Primer sequences for *IDH1*-R132 LNA-LAMP

LAMP primer	Sequence
F3	5′-TGTGGAAATCACCAAATGG-3′
B3	5′-GCCATGAAAAAAAAAACATGC-3′
FIP	5′-CGGGGGATATTTTTGCAGATAATGG*tttt*CACCATACGAAATATTCTGGGT 3′
BIP	5′-GTGGATGGGTAAAACCTATCATCATAG*tttt*CACATTATTGCCAACATGACT 3′
SALP	5′-*gatc*GT**XXX**CATGCTTATGGGGGATC 3′

The position of LNAs and DNA sequence within the SALP vary based on the R132 variant targeted, where **XXX** represents the codon sequence of each respective *IDH1*-R132 variant. Specific sequences and LNA locations for variants are specified in Table 3.

### Colorimetric LNA-LAMP

LNA-LAMP was performed using WarmStart^®^ Colorimetric LAMP Master Mix (New England Biolabs, NEB), custom LAMP primers containing LNAs (IDT), and 1M betaine (Sigma-Aldrich). LAMP reactions were performed in nuclease-free PCR tubes at 25 or 50 µl reaction volumes. Primer concentrations were 0.2 µM F3, 0.2 µM B3, 1.6 µM FIP, 1.6 µM BIP, and 0.8 µM LNA-SALP. Reactions were optimized by incubation at temperatures ranging from 67 to 69°C for 35–45 min and then denatured at 95°C for 2 min. A final reaction temperature of 68°C was identified as the optimal temperature for the final selection of primers. Amplification was confirmed via 4% agarose gel (EtBr) electrophoresis at 100 volts for 1 h and then imaged with a BioRad ChemiDoc MP™ Imaging System.

### Endpoint colorimetric LNA-LAMP

LAMP reactions were prepared at 50 µl volumes, incubated at the conditions specified above, and then cooled to 4°C. Reactions were then transferred to a clear flat-bottom 96-well plate. Colorimetric changes were measured using absorbance at 430 and 560 nm in a Synergy H1 plate reader. Absorbance ratios (A430/A560) were then calculated.

### Synthetic DNA for IDH1 variants R132 wild-type, R132H, R132G, R132S, R132L, and R132C

Six hundred and fifty-three base pair synthetic DNA fragments (gBlocks^®^) containing the region with the SNV were purchased from IDT. Unless otherwise specified, 1.0 × 10^5^ copies of synthetic DNA were added to each 50-µl reaction, while 5.0 × 10^4^ copies were added to each 25-µl reaction. Sequences of gBlocks^®^ for each variant are listed below. Bolded and highlighted sequences represent the location targeted by LNA-based SALPs.

#### IDH1-R132 wild-type gBlock^®^ sequence

CTATGATTTAGGCATAGAGAATCGTGATGCCACCAACGACCAAGTCACCAAGGATGCTGCAGAAGCTATAAAGAAGCATAATGTTGGCGTCAAATGTGCCACTATCACTCCTGATGAGAAGAGGGTTGAGGAGTTCAAGTTGAAACAATGTGGAAATCACCAAATGGCACCATACGAAATATTCTGGGTGGCACGGTCTTCAGAGAAGCCATTATCTGCAAAAATATCCCCCGGCTTGTGAGTGGATGGGTAAAACCTATCATCATAGGTC**GT**CATGCTTATGGGGATCAAGTAAGTCATGTTGGCAATAATGTGATTTTGCATGTTTTTTTTTTCATGGCCCAGAAATTTCCAACTTGTATGTGTTTTATTCTTATCTTTTGGTATCTACACCCATTAAGCAAGGTATGAAATTGAGAAATGCATATATGTATAACTGTATATTTACACACATTTAGCTAAAGGCAAATACAAATAAACTTACAAATAGGCGTCCATCTCAACACATTTTTTTTAAACATGCTGTTTTTTTTCCTTTATCCTTTTATTCAGTTATACCATATGATATTGCCATTTTTATGTTGGTAATTTCATATGGTTCAACCAGATCTGTGGTTTTCAACACTGGCTGCACAATAGGATCCCCTTACAAG.

#### IDH1-R132H gBlock^®^ sequence

CTATGATTTAGGCATAGAGAATCGTGATGCCACCAACGACCAAGTCACCAAGGATGCTGCAGAAGCTATAAAGAAGCATAATGTTGGCGTCAAATGTGCCACTATCACTCCTGATGAGAAGAGGGTTGAGGAGTTCAAGTTGAAACAATGTGGAAATCACCAAATGGCACCATACGAAATATTCTGGGTGGCACGGTCTTCAGAGAAGCCATTATCTGCAAAAATATCCCCCGGCTTGTGAGTGGATGGGTAAAACCTATCATCATAGGT**CA**TCATGCTTATGGGGATCAAGTAAGTCATGTTGGCAATAATGTGATTTTGCATGTTTTTTTTTTCATGGCCCAGAAATTTCCAACTTGTATGTGTTTTATTCTTATCTTTTGGTATCTACACCCATTAAGCAAGGTATGAAATTGAGAAATGCATATATGTATAACTGTATATTTACACACATTTAGCTAAAGGCAAATACAAATAAACTTACAAATAGGCGTCCATCTCAACACATTTTTTTTAAACATGCTGTTTTTTTTCCTTTATCCTTTTATTCAGTTATACCATATGATATTGCCATTTTTATGTTGGTAATTTCATATGGTTCAACCAGATCTGTGGTTTTCAACACTGGCTGCACAATAGGATCCCCTTACAAG.

#### IDH1-R132G gBlock^®^ sequence

CTATGATTTAGGCATAGAGAATCGTGATGCCACCAACGACCAAGTCACCAAGGATGCTGCAGAAGCTATAAAGAAGCATAATGTTGGCGTCAAATGTGCCACTATCACTCCTGATGAGAAGAGGGTTGAGGAGTTCAAGTTGAAACAATGTGGAAATCACCAAATGGCACCATACGAAATATTCTGGGTGGCACGGTCTTCAGAGAAGCCATTATCTGCAAAAATATCCCCCGGCTTGTGAGTGGATGGGTAAAACCTATCATCATAGGT**GG**TCATGCTTATGGGGATCAAGTAAGTCATGTTGGCAATAATGTGATTTTGCATGTTTTTTTTTTCATGGCCCAGAAATTTCCAACTTGTATGTGTTTTATTCTTATCTTTTGGTATCTACACCCATTAAGCAAGGTATGAAATTGAGAAATGCATATATGTATAACTGTATATTTACACACATTTAGCTAAAGGCAAATACAAATAAACTTACAAATAGGCGTCCATCTCAACACATTTTTTTTAAACATGCTGTTTTTTTTCCTTTATCCTTTTATTCAGTTATACCATATGATATTGCCATTTTTATGTTGGTAATTTCATATGGTTCAACCAGATCTGTGGTTTTCAACACTGGCTGCACAATAGGATCCCCTTACAAG.

#### IDH1-R132S gBlock^®^ sequence

CTATGATTTAGGCATAGAGAATCGTGATGCCACCAACGACCAAGTCACCAAGGATGCTGCAGAAGCTATAAAGAAGCATAATGTTGGCGTCAAATGTGCCACTATCACTCCTGATGAGAAGAGGGTTGAGGAGTTCAAGTTGAAACAATGTGGAAATCACCAAATGGCACCATACGAAATATTCTGGGTGGCACGGTCTTCAGAGAAGCCATTATCTGCAAAAATATCCCCCGGCTTGTGAGTGGATGGGTAAAACCTATCATCATAGGT**AG**TCATGCTTATGGGGATCAAGTAAGTCATGTTGGCAATAATGTGATTTTGCATGTTTTTTTTTTCATGGCCCAGAAATTTCCAACTTGTATGTGTTTTATTCTTATCTTTTGGTATCTACACCCATTAAGCAAGGTATGAAATTGAGAAATGCATATATGTATAACTGTATATTTACACACATTTAGCTAAAGGCAAATACAAATAAACTTACAAATAGGCGTCCATCTCAACACATTTTTTTTAAACATGCTGTTTTTTTTCCTTTATCCTTTTATTCAGTTATACCATATGATATTGCCATTTTTATGTTGGTAATTTCATATGGTTCAACCAGATCTGTGGTTTTCAACACTGGCTGCACAATAGGATCCCCTTACAAG.

#### IDH1-R132L gBlock^®^ sequence

CTATGATTTAGGCATAGAGAATCGTGATGCCACCAACGACCAAGTCACCAAGGATGCTGCAGAAGCTATAAAGAAGCATAATGTTGGCGTCAAATGTGCCACTATCACTCCTGATGAGAAGAGGGTTGAGGAGTTCAAGTTGAAACAATGTGGAAATCACCAAATGGCACCATACGAAATATTCTGGGTGGCACGGTCTTCAGAGAAGCCATTATCTGCAAAAATATCCCCCGGCTTGTGAGTGGATGGGTAAAACCTATCATCATAGGT**CT**TCATGCTTATGGGGATCAAGTAAGTCATGTTGGCAATAATGTGATTTTGCATGTTTTTTTTTTCATGGCCCAGAAATTTCCAACTTGTATGTGTTTTATTCTTATCTTTTGGTATCTACACCCATTAAGCAAGGTATGAAATTGAGAAATGCATATATGTATAACTGTATATTTACACACATTTAGCTAAAGGCAAATACAAATAAACTTACAAATAGGCGTCCATCTCAACACATTTTTTTTAAACATGCTGTTTTTTTTCCTTTATCCTTTTATTCAGTTATACCATATGATATTGCCATTTTTATGTTGGTAATTTCATATGGTTCAACCAGATCTGTGGTTTTCAACACTGGCTGCACAATAGGATCCCCTTACAAG.

#### IDH1-R132C gBlock^®^ sequence

CTATGATTTAGGCATAGAGAATCGTGATGCCACCAACGACCAAGTCACCAAGGATGCTGCAGAAGCTATAAAGAAGCATAATGTTGGCGTCAAATGTGCCACTATCACTCCTGATGAGAAGAGGGTTGAGGAGTTCAAGTTGAAACAATGTGGAAATCACCAAATGGCACCATACGAAATATTCTGGGTGGCACGGTCTTCAGAGAAGCCATTATCTGCAAAAATATCCCCCGGCTTGTGAGTGGATGGGTAAAACCTATCATCATAGGT**TG**TCATGCTTATGGGGATCAAGTAAGTCATGTTGGCAATAATGTGATTTTGCATGTTTTTTTTTTCATGGCCCAGAAATTTCCAACTTGTATGTGTTTTATTCTTATCTTTTGGTATCTACACCCATTAAGCAAGGTATGAAATTGAGAAATGCATATATGTATAACTGTATATTTACACACATTTAGCTAAAGGCAAATACAAATAAACTTACAAATAGGCGTCCATCTCAACACATTTTTTTTAAACATGCTGTTTTTTTTCCTTTATCCTTTTATTCAGTTATACCATATGATATTGCCATTTTTATGTTGGTAATTTCATATGGTTCAACCAGATCTGTGGTTTTCAACACTGGCTGCACAATAGGATCCCCTTACAAG.

### Negative-template control DNA

*Pseudomonas aeruginosa* DNA was extracted from inoculated LB broth and purified using the QIAamp DNA Mini Kit protocol (Qiagen) for use as a negative-template control.

### Archived tumor samples

Glioma tumor samples with known *IDH1*-R132 mutational status were provided by Advocate Aurora Research Institute, LLC, Milwaukee, WI (Aurora IRB# 14–79). Samples were prepared as previously described [1] and diluted to a 1:100 ratio in molecular-grade water. Ten microliter of 1:100 dilutions were utilized for each 50-µl reaction.

### Statistical analyses

Due to small sample sizes which render the underlying assumptions of parametric tests such as a *t*-test questionable, we chose to employ more conservative, nonparametric methods. A nonparametric two-sample Wilcoxon test with Bonferroni-corrected *P* values was performed for data found in 6C, with conclusions graphically represented in Fig. 6D. The number of observations for each group along with test statistics is available in Supplementary Table S2.

## Results

### Requirements of LNA-mediated specificity vary based on the SNV position and composition of neighboring nucleotides

**Figure 3:**
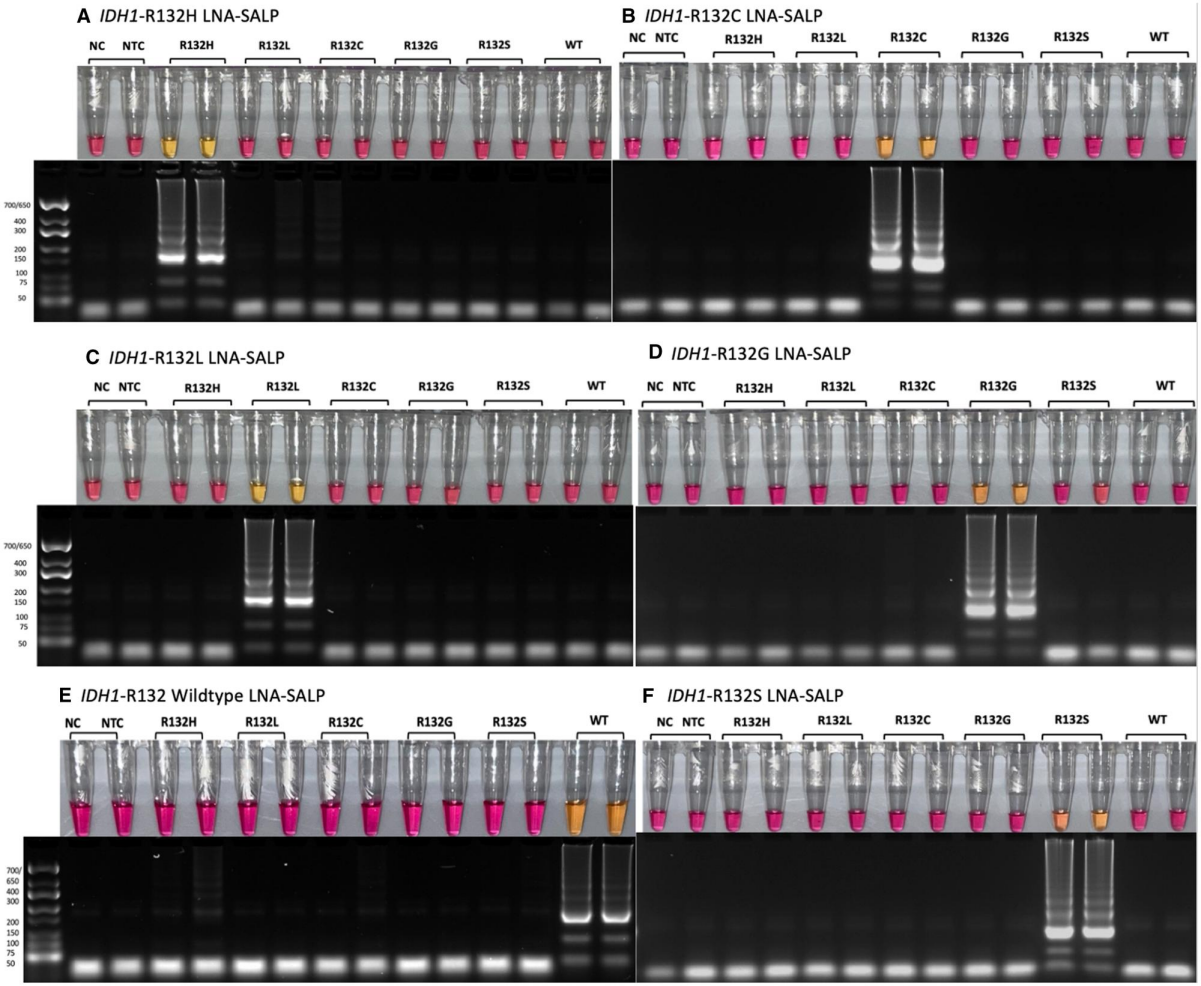
(**A–F**) LNA-SALPs targeting *IDH1*-R132 variants mediate allelic specificity. The sample order is as follows: 1. NC (molecular grade H_2_O), 2. NTC (*P. aeruginosa* DNA), 3 and 4. *IDH1*-R132H DNA at 5.0 × 10^4^ copies, 5 and 6. *IDH1*-R132L DNA at 5.0 × 10^4^ copies, 7 and 8. *IDH1*-R132C DNA at 5.0 × 10^4^ copies, 9 and 10. *IDH1*-R132G DNA at 5.0 × 10^4^ copies, 11 and 12. *IDH1*-R132S DNA at 5.0 × 10^4^ copies, 13 and 14. *IDH1*-R132 wild-type DNA at 5.0 × 10^4^ copies. Reactions were incubated at 68°C for approximately 35 min. These results are representative of multiple experiments.

During the initial optimization steps, we integrated a single LNA into the SALPs to target each variant’s respective altered base (Table 1). To screen for sequence specificity, primers targeting each variant were tested using the synthetic DNA from each of the five *IDH1*-R132 variants and wild-type *IDH1*. We found that for the variants with a mutation at the second codon position (R132H and R132L), a single LNA at the SNV was sufficient to mediate specificity. However, a single LNA did not impart specificity for the first-position variants (R132C, R132G, and R132S) (Supplementary Fig. S1). We subsequently added a second LNA at the base preceding the SNV, which imparted impressive specificity for second-position variants (Fig. 3A and C). However, this arrangement of LNAs within the SALP did not provide specificity for the detection of first-position variants or *IDH1*-R132 wild-type (Supplementary Fig. S2A and S2B). Next, we added a third LNA to encompass the entire R132 codon. We found that this resulted in a lack of specificity for all R132 variants tested and, in some cases, poor amplification efficiency (Supplemental Fig. S3A and S3B).

To achieve LNA-mediated SNV specificity for *IDH1*-R132 variants, we next incorporated two LNAs targeting the SNV and its adjacent 3′-base. We found that this LNA-containing primer did not impart specificity for second-position variants (Supplementary Fig. S4) but did for first-position variants and wild-type (Fig. 3B–F). Thus, the final selection of LNA-SALPs included the LNA at codon positions 1 and 2 for first- and second-position variants and codon positions 2 and 3 for wild-type (Table 3, Fig. 3A–F). In all cases, reactions were visually interpreted after approximately 35 min following a 68°C incubation. Agarose gel electrophoresis results corresponded with colorimetric changes and showed no evidence of background amplification of non-complementary DNA, further supporting the specificity of LNA-LAMP (Fig. 3A–F).

**Table 3.** The genetic sequence found at the R132 codon of wild-type *IDH1* and each respective *IDH1*-R132 variant targeted in this study

*IDH1*-R132 variant	Codon position of SNV	Codon sequence
Wild-type	N/A	CGT
R132C	1	**T**GT
R132G	1	**G**GT
R132S	1	**A**GT
R132H	2	C**A**T
R132L	2	C**T**T

Highlighted sequences represent the nucleotides targeted by complementary LNAs to mediate specificity, while bolded nucleotides represent the SNV.

### Characterization of *IDH1*-R132 LNA-LAMP limit of detection

**Figure 4:**
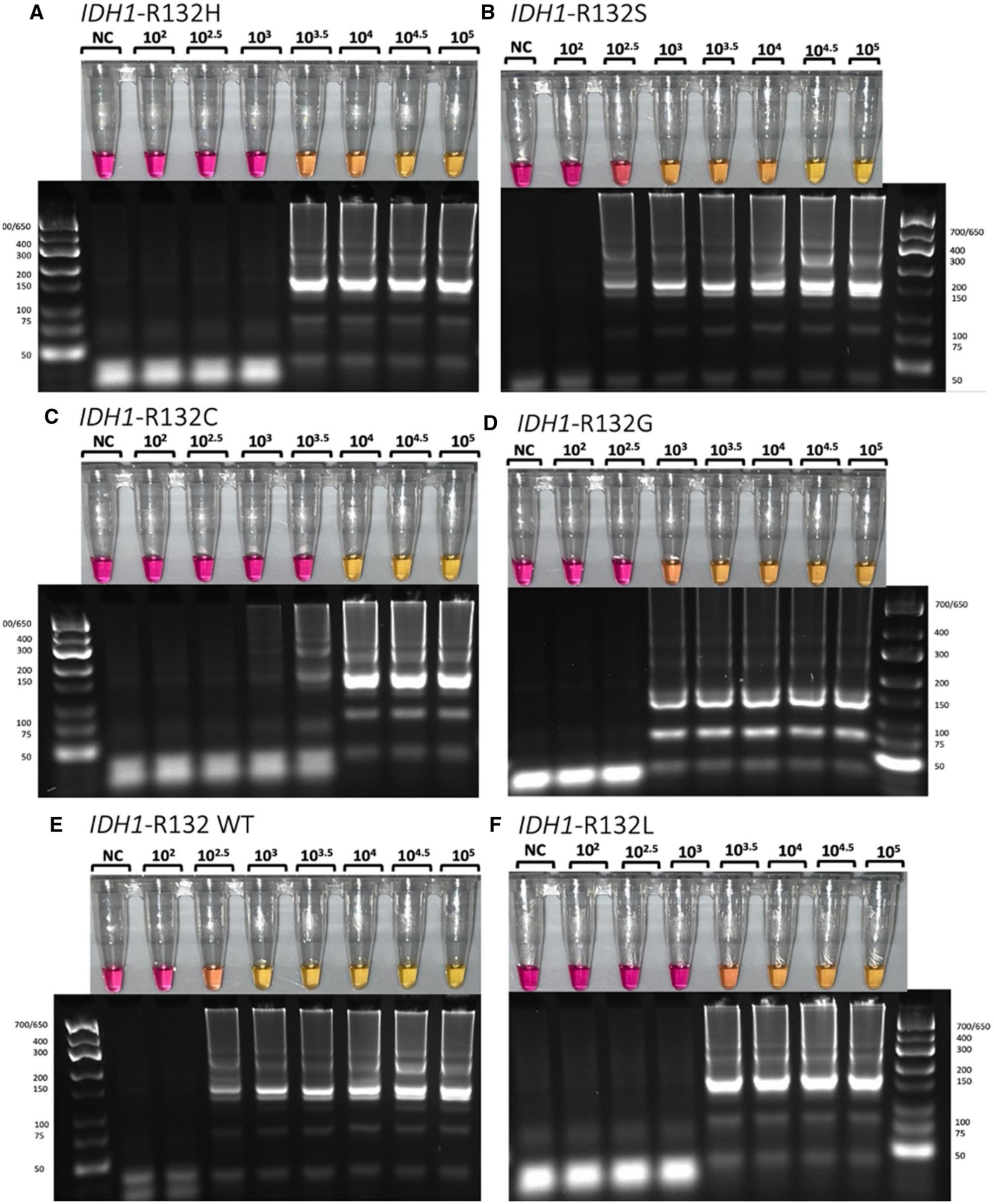
(**A–F**) Serial dilutions of *IDH1*-R132 DNA complementary to LNA-SALPs for each corresponding variant demonstrate that 5.0 × 10^4^ copies elicit reliable colorimetric changes. DNA complementary to each variant’s primer was titrated with concentrations ranging from 5.0 × 10^2^ copies to 5.0 × 10^5^ copies per 25 µl reaction volume. Reactions were incubated at 68°C for 45 min. 5.0 × 10^4^ copies were reproducibly detected colorimetrically for each of the *IDH1*-R132 SNVs.

To further characterize the assay using the selected LNA-SALPs, we next sought to determine the optimal DNA input to achieve consistent amplification for all variants while maintaining SNV discrimination. To this end, we titrated copy number input in 0.5× intervals with values ranging from 5.0 × 10^2^ to 5.0 × 10^5^. We identified 5.0 × 10^4^ as the copy number able to consistently amplify all variants (Fig. 4A–F). Thus, we aimed for a copy number input of 5.0 × 10^4^ in all subsequent experiments.

### Use of absorbance spectroscopy to evaluate colorimetric LNA-LAMP results

**Figure 5:**
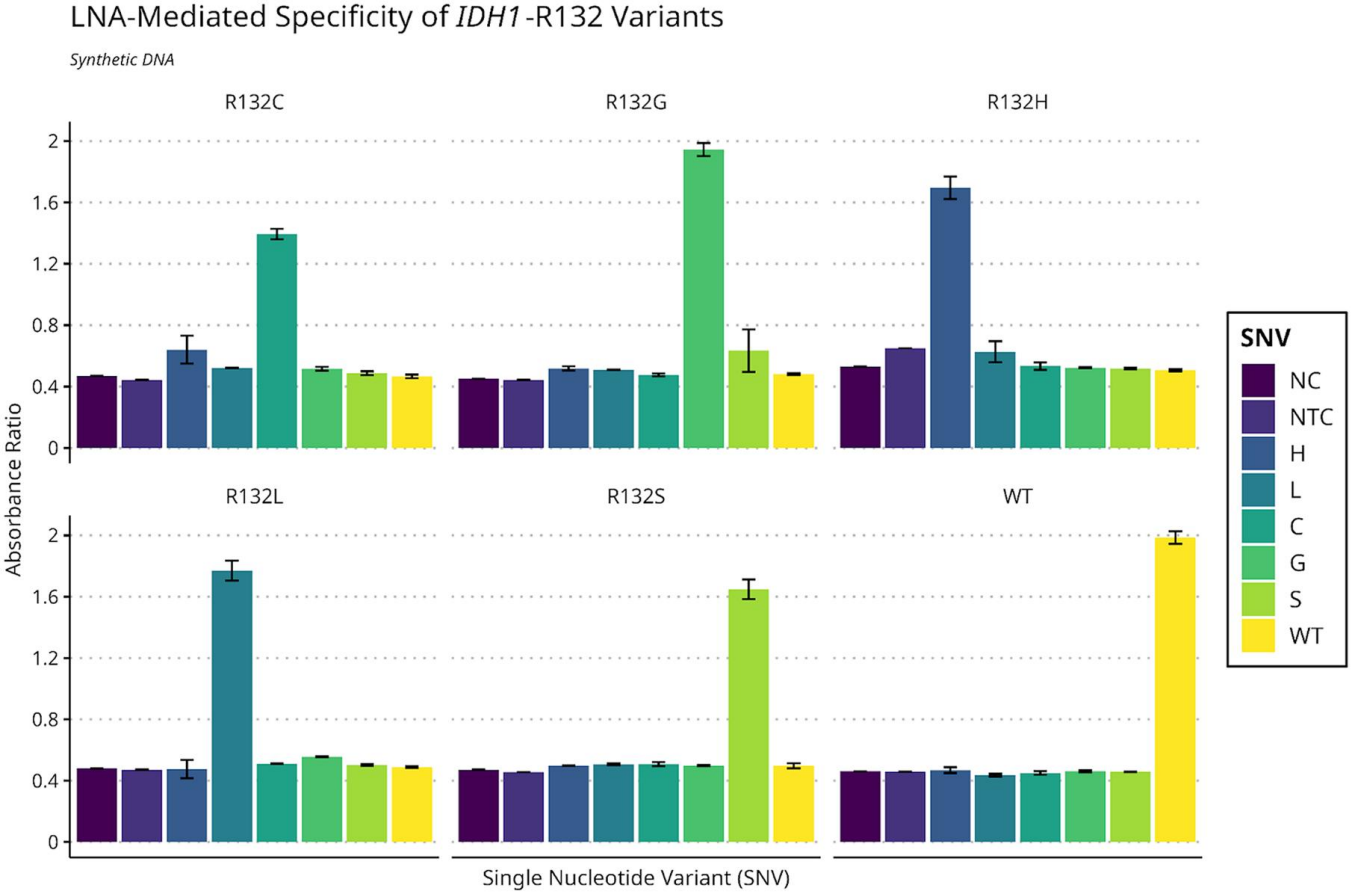
Results from the *IDH1*-R132 genotyping panel can be interpreted using absorbance ratio as an endpoint analysis. For each respective primer, the negative control (NC) consisted of molecular grade H_2_O, while the NTC consisted of *P. aeruginosa* DNA. All reactions for absorbance-based work were 50-µl reactions, and 1.0 × 10^5^ copies of synthetic DNA were added in duplicate for all R132 variants. Each bar is an average of two data points, and these results are representative of multiple experiments.

To eliminate the subjectivity of interpreting colorimetric LAMP results and provide an objective numerical readout, we assessed the feasibility of utilizing absorbance ratios at wavelengths 430 nm/560 nm. Endpoint analysis was performed by transferring completed colorimetric LAMP reactions to a clear, flat-bottomed 96-well plate and measuring the absorbance. Only the reactions containing synthetic DNA with the SNV complementary to the allele-specific LNA-SALP resulted in an increased absorbance ratio above 0.8 (Fig. 5). The average absorbance ratio for all positive results is 1.7 ± 0.43, while the ratio for negative results is 0.51 ± 0.12. The average absorbance ratio of nontemplate controls is 0.49 ± 0.13. Importantly, we found that the absorbance ratios for nontarget sequences closely resembled those determined for negative controls. Collectively, these findings demonstrate the feasibility of using absorbance ratio to objectively interpret the results of an allele-specific genotyping panel with colorimetric LNA-LAMP.

### LNA-LAMP specifically detects the *IDH1*-R132H variant in glioma tumor lysates

Due to the ability of LAMP to amplify target DNA in the presence of a complex biological matrix [1], we next sought to determine if LNA-LAMP could provide specificity using an input of minimally processed glioma tumors. We first screened the ability of LNA-LAMP to amplify lysates when paired with an *IDH1*-wild-type-specific primer. Since IDH1-R132 mutations are heterozygous [8], all samples amplified as expected (Fig. 6A and Supplementary Fig. S5).

**Figure 6:**
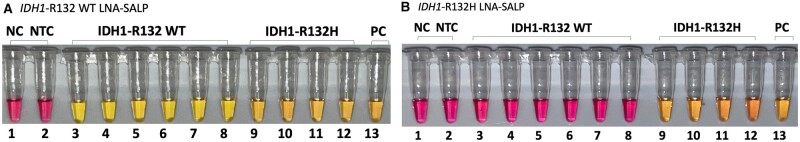
(**A**) LNA-LAMP amplifies target DNA using glioma tumor lysates. Reactions were prepared with LNA-SALPs targeting *IDH1*-R132 wild-type. The sample order is as follows: 1. NC (molecular grade H_2_O), 2. NTC (*P. aeruginosa* DNA), 3–8 *IDH1*-R132 wild-type glioma tumor lysates, 9–12 *IDH1*-R132H mutant glioma tumor lysates, 13. *IDH1*-R132 wild-type synthetic DNA at 1.0 × 10^6^ copies. Absorbance ratios for these samples can be found in Supplementary Fig. S5. These results are representative of multiple experiments. (**B**) LNA-LAMP specifically detects *IDH1*-R132H in glioma tumor lysates. Reactions were prepared with LNA-SALPs targeting *IDH1*-R132H. The sample order is as follows: 1. NC (molecular grade H_2_O), 2. NTC (*P. aeruginosa* DNA), 3–8. *IDH1*-R132 wild-type glioma tumor lysates, 9–12 *IDH1*-R132H mutant glioma tumor lysates, 13. *IDH1*-R132H synthetic DNA at 1.0 × 10^6^ copies. These results are representative of multiple experiments. **(C**) Absorbance ratios of IDH1-R132H LNA-LAMP reactions using glioma tumor lysates. Colorimetric changes and sample input correspond to those found in Fig. 6B. The height of the bar plots and points correspond to the mean and observed absorbance ratios for each lysate, respectively. **(D**) Grouped results of *IDH1*-R132H LNA-LAMP absorbance ratios based on glioma mutational status. Here we show the results of pairwise comparisons of mean absorbance ratios across the four sample groups shown in Fig. 6C: negative control, *IDH1*-R132 wild-type, *IDH1*-R132H mutant, and positive control. Due to the relatively small number of samples in each group, we defaulted to using a nonparametric two-sample Wilcoxon test with Bonferroni-corrected *P*-values. The number of observations for each group as well as test statistics are available in Supplementary Table 2

**Figure 6:**
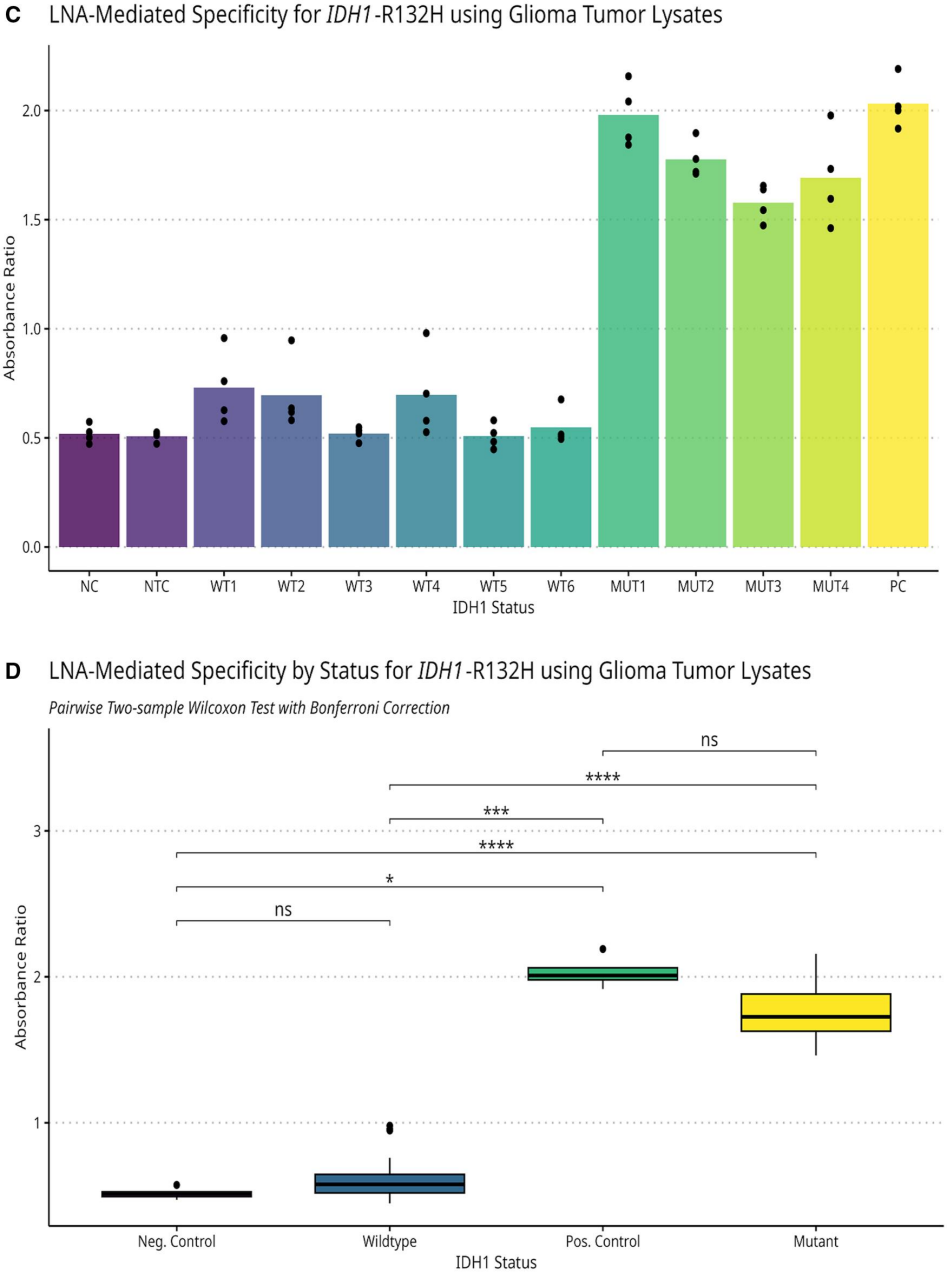
(Continued).

Next, we sought to determine if LNA-LAMP could selectively amplify lysates known to contain the *IDH1*-R132H mutation. When paired with an R132H-specific LNA-SALP, successful allelic discrimination occurred, even in cases where the *IDH1*-R132H mutational status was limited to a subpopulation of the tumor (Fig. 6B and Supplementary Table S1). All reactions containing *IDH1*-R132H lysates were visually positive within 30–34 min. All *IDH1* wild-type samples were visually negative for amplification. We then compared absorbance ratios for *IDH1*-R132 wild-type and mutant samples (Fig. 6C). For *IDH1*-R132H mutant lysates, the results are unambiguous: compared to the negative control and wild-type group, absorbance levels are significantly higher (*P* < 0.001) for both. Comparing the positive control to both negative control and wild-type, there is a significant difference at the *P* < 0.05 and *P* < 0.001 levels, respectively (Fig. 6D). Importantly, no significant difference in absorbance ratios is observed between wild-type and negative controls or between the *IDH1*-R132H mutant samples and positive controls. Additional information regarding statistical analysis can be found in Supplementary Table S2. Repeat testing with the remaining R132 variant-specific LNA-SALPs suggested that no nonspecific amplification occurred (Supplementary Figs S6A–S6D and S7).

## Discussion

In this study, we developed a genotyping panel utilizing customized LNA-SALPs capable of single nucleotide discrimination of *IDH1*-R132 variants. This method rapidly and specifically detects the five most common clinically relevant *IDH1*-R132 SNVs. Optimizing the position and number of LNAs within the SALP respective to each SNV allowed us to identify sets of primers capable of detecting all targeted *IDH1*-R132 variants utilizing a single reaction temperature. We then adapted the assay to an endpoint format readout using an absorbance ratio to quantify colorimetric results, providing a more objective measure of a positive reaction. Next, we established the feasibility of using LNA-LAMP to specifically detect SNVs in glioma tumor lysates. Importantly, no evidence of nonspecific amplification was detected.

We determined that the number and position of LNAs necessary for specificity varied based on the position of the SNV in the R132 codon. Of note, in some instances, there was evidence of amplification that was not of sufficient magnitude to induce a colorimetric change. Hence, those LNA-SALPs were eliminated from use (Supplementary Figs S1–S4). As a result, additional LNA-SALPs were designed, tested, and selected (Fig. 3A–F). Interestingly, for the variants in which the mutation occurs in the second position of the codon (R132H and R132L), a single LNA complementary to the SNV was sufficient to mediate specificity; however, this was not the case with the remaining variants. The specific detection of first-position variants (R132C, R132G, R132S) and wild-type required an additional LNA complementary to the SNV and subsequent 3′-base. Previous findings showed cross-reactivity between SNVs when using an LNA-SALP [7]. While this group used a single LNA in the SALP targeting the SNV, here we demonstrate that the addition of a second LNA adjacent to the SNV improves specificity when the reaction is halted at the appropriate cutoff time. It is important to note that, like most DNA amplification methods, all samples will amplify if incubated in excess, despite the initial specificity granted by the LNAs. Thus, determining the earliest possible cutoff time where the target samples are unambiguously positive and nontarget samples are negative is necessary. We found this cutoff time to occur at approximately 35 min. We also found that the addition of each LNA increased the optimal reaction temperature requirement by 1°C. This may explain the lack of success when using LNAs encompassing the entire codon, as raising the temperature to 69°C exceeds the optimal range for *Bst* polymerase 2.0 (66–68°C) [9]. However, we did note that LNA-LAMP reactions were faster at 68°C and when performed with a 50-µl reaction volume. Furthermore, we found the addition of 5.0 × 10^4^ copies of *IDH1*-R132 per 25-µl reaction volume to elicit the most reliable and timely colorimetric change for each SNV tested (Fig. 4A–F). As tumor tissue contains approximately 10^8^–10^9^ cells per gram, 5.0 × 10^4^ copies is the equivalent DNA in 1/10,000th of a gram [10]. Using this principle as a basis for the preparation and subsequent dilution of tumor lysates, we did not see any false positives or negatives in LNA-LAMP, or in our previous work with PNA-LAMP [1].

Since *IDH1*-R132 mutations are heterozygous and tumors are typically heterogeneous [11], there were likely fewer copies of *IDH1*-R132H in glioma lysates than copies of wild-type *IDH1*-R132. This may explain the stronger colorimetric change observed visually and greater absorbance ratios when using LNA-LAMP specific to wild-type *IDH1* (Fig. 5A and Supplementary Fig. S5). Despite this, we demonstrate that LNA-LAMP can specifically detect target SNVs when they are the minor population present in a clinical sample. Additionally, the use of a SALP designed to target *IDH1*-R132 wild-type serves as an amplification control. Since LNA-LAMP can be used with minimally processed lysates, it is also an ideal candidate for time-sensitive genotyping. Though we were unable to acquire tumor samples with *IDH1*-R132 mutations other than R132H, we are confident that this method would successfully detect other variants. We recognize that additional experimentation will be necessary to establish the appropriate absorbance threshold for the determination of mutational status; however, we did not have the sample population required. We also recognize that assessing the ability of this method to accurately detect low-frequency alleles needs to be further explored and compared to current methods such as Sanger sequencing, which has a variant allele frequency (VAF) of 20%, and next-generation sequencing with a VAF of 5%–10%. In comparison with similar DNA amplification methods, LAMP has demonstrated a 10-fold greater sensitivity than traditional PCR but less sensitivity than qPCR and nested PCR [12]. While PCR can also function with an input of tissue lysates [13], the lack of thermocycling and time to completion for LAMP may make it a convenient choice, particularly for low-resource or time-sensitive applications.

In this study, we utilized *IDH1* as a target because it is an important biomarker in gliomas. We envision the use of LNA-LAMP to develop genotyping panels that detect other clinically relevant SNVs. The use of absorbance ratios to determine the results of colorimetric LNA-LAMP could be adapted to a more automated format, where an instrument is able to both maintain the necessary temperature requirements and collect absorbance data thereafter, thus negating the necessity of transferring samples upon reaction completion. A more automated workflow such as this could be particularly useful in cases where results are time-sensitive, which would be further aided by the rapid nature of LNA-LAMP and its ability to analyze tissue without preanalytical nucleic acid extraction steps.

## Supplementary Material

bpae012_Supplementary_Data

## Data Availability

The data underlying this article are available in the article and in its online supplementary material.
