# Supplementary material for: Rapid IDH1-R132 genotyping panel utilizing locked nucleic acid loop-mediated isothermal amplification
Source: Biol Methods Protoc. 2024 Feb 21;9(1):bpae012. doi: 10.1093/biomethods/bpae012 (PMC10984729; doi:10.1093/biomethods/bpae012)
Supplement: bpae012_Supplementary_Data [file bpae012_supplementary_data.pdf]

# Supplemental figures

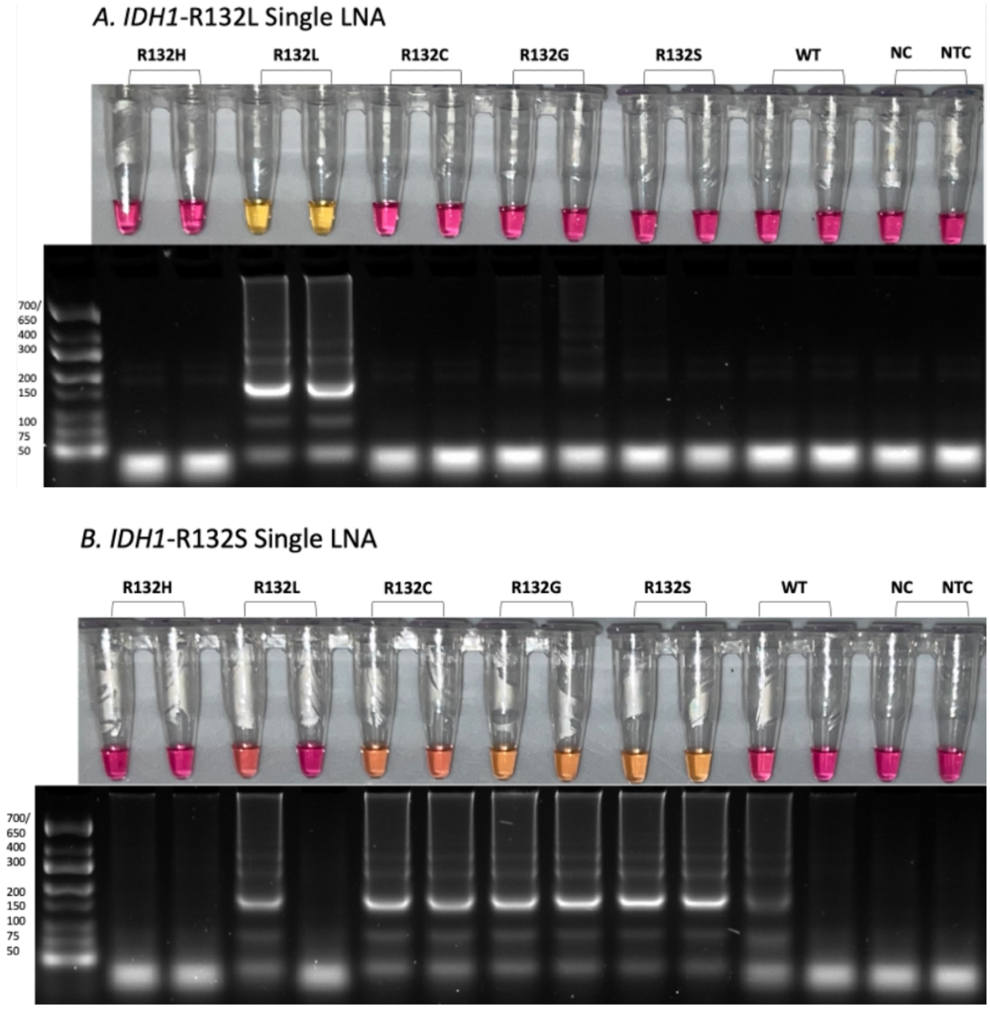


**Supplemental Figure 1A-1B. A single LNA at the SNV mediates specificity only for second-position variants.** Sample order is as follows: 1-2. *IDH1*-R132H DNA at $5.0X10^4$ copies, 3-4. *IDH1*-R132L DNA at $5.0X10^4$ copies, 5-6. *IDH1*-R132C DNA at $5.0X10^4$ copies, 7-8. *IDH1*-R132G DNA at $5.0X10^4$copies, 9-10. *IDH1*-R132S DNA at $5.0X10^4$ copies, 11-12 *IDH1*-R132 wildtype DNA at $5.0X10^4$ copies, 13. NC (molecular grade $H_2O$), 14. NTC (*P. aeruginosa* DNA). Reactions were incubated at 67$^o$C for approximately 35 minutes. These results are representative of multiple experiments. Similar results to those shown in supplemental figure 1A are found when assaying the other second position variant, *IDH1*-R132H, with a single LNA at the SNV. Similarly, results shown in supplemental figure 1B are representative of those found when assaying first position variants.

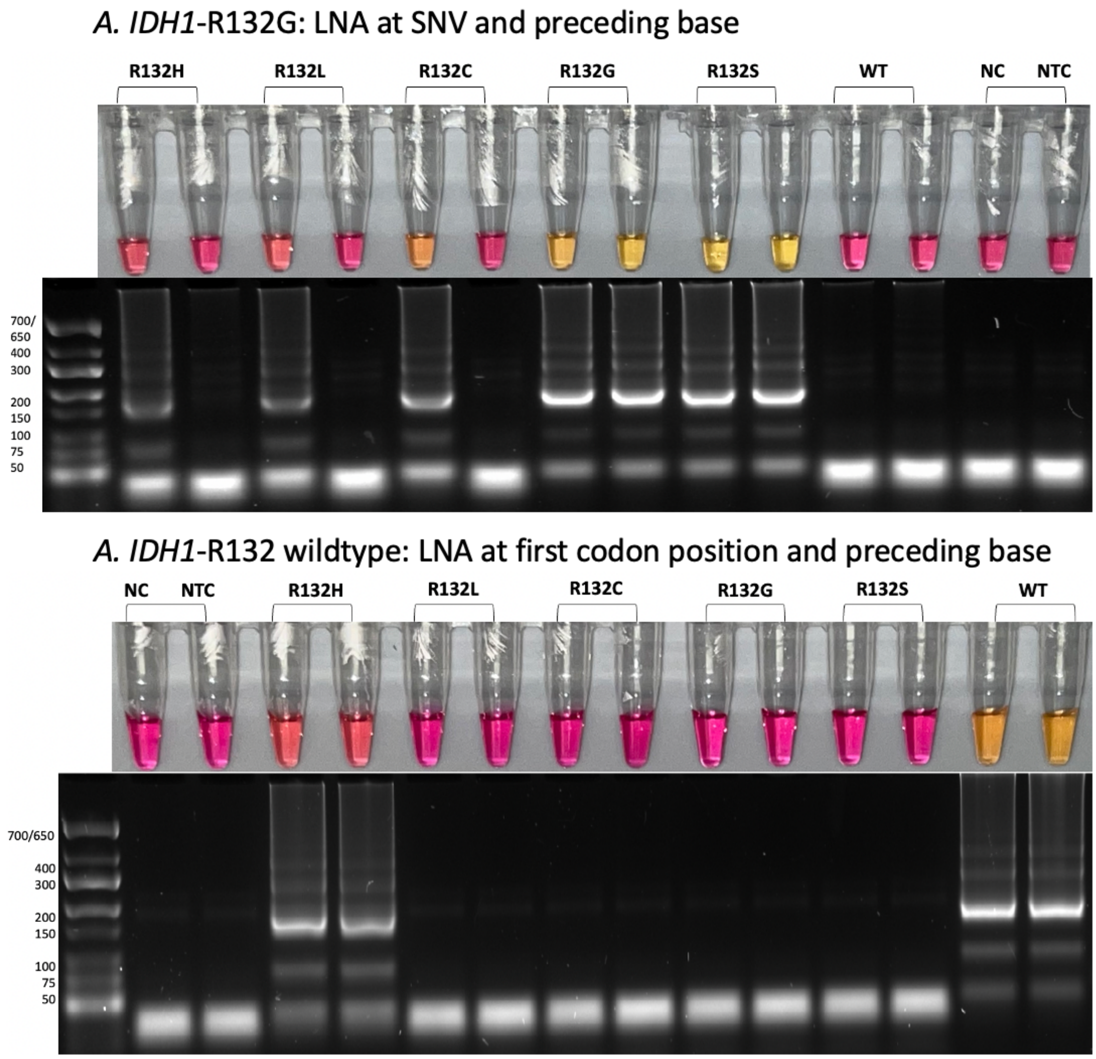


**Supplemental Figure 2A-2B. Two LNAs at the first two positions of the R132G and IDH1-R132 wildtype codon are unable to mediate allele specificity**. Sample order is as follows: Sample order is as follows: 1-2. *IDH1*-R132H DNA at $5.0X10^4$ copies, 3-4. *IDH1*-R132L DNA at $5.0X10^4$ copies, 5-6. *IDH1*-R132C DNA at $5.0X10^4$ copies, 7-8. *IDH1*-R132G DNA at $5.0X10^4$copies, 9-10. *IDH1*-R132S DNA at $5.0X10^4$ copies, 11-12 *IDH1*-R132 wildtype DNA at $5.0X10^4$ copies, 13. NC (molecular grade $H_2O$), 14. NTC (*P. aeruginosa* DNA). Reactions were incubated at 68°C for approximately 35 minutes. These results are representative of multiple experiments. Similar results to those shown in supplemental figure 2A are found when assaying other first position variants with LNAs at codon positions 1 and 2.

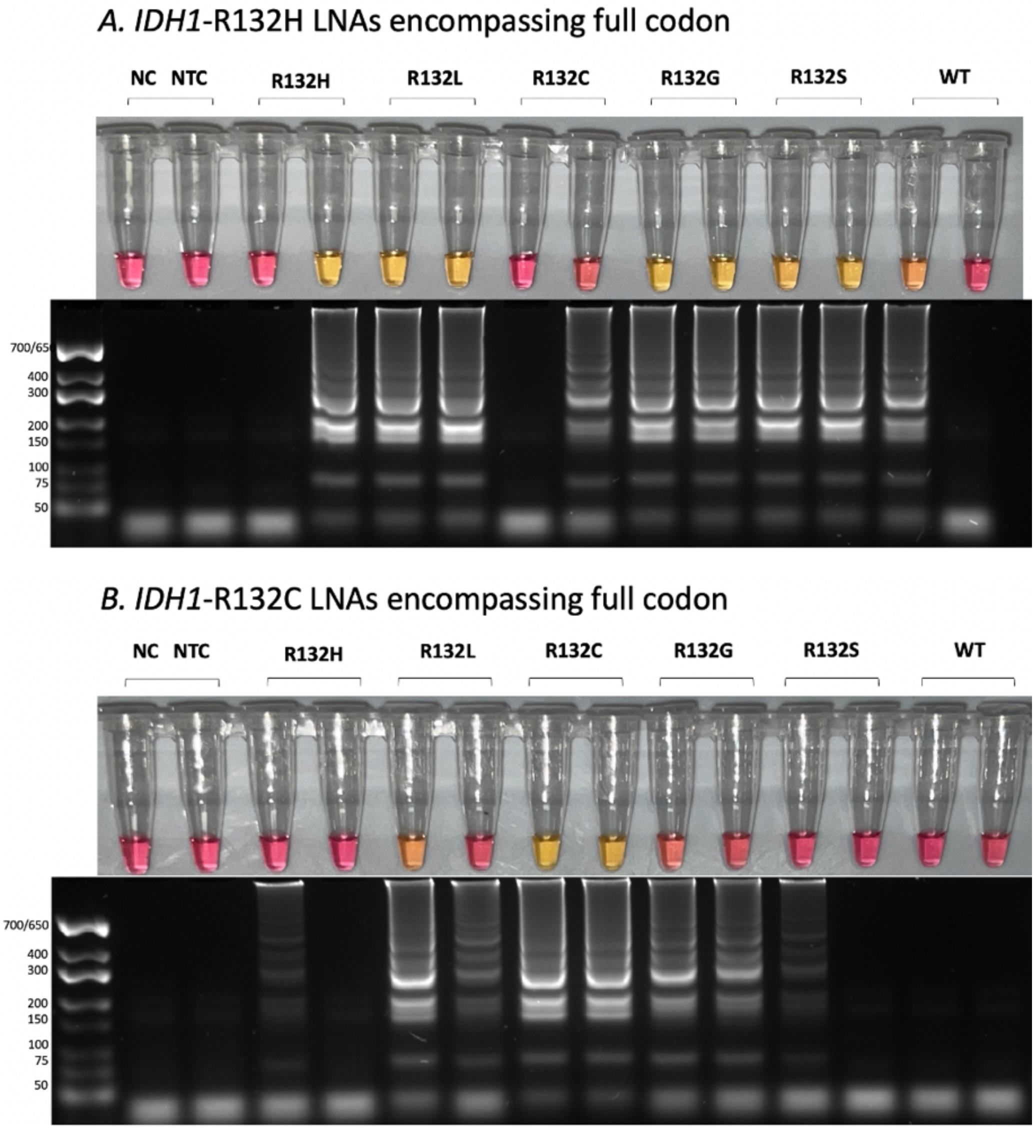


**Supplemental Figure 3A-3B. Fully locking the codon lowers reaction efficiency and decreases specificity for all IDH1-R132 variants.** Sample order is as follows: 1. NC (molecular grade $H_2O$), 2. NTC (*P. aeruginosa*), 3-4. *IDH1*-R132H DNA at 5.0X$10^4$ copies, 5-6. *IDH1*-R132L DNA at 5.0X$10^4$ copies, 7-8. *IDH1*-R132C DNA at 5.0X$10^4$ copies, 9-10. *IDH1*-R132G DNA at 5.0X$10^4$copies, 11-12. *IDH1*-R132S DNA at 5.0X$10^4$ copies, 13-14 *IDH1*-R132 wildtype DNA at 5.0X$10^4$ copies. Reactions were incubated at 69$^o$C for approximately 40 minutes. These results are representative of multiple experiments. Similar results to those shown in supplemental figure 3A-3B are found when assaying all variants, regardless of the codon position of the SNV.

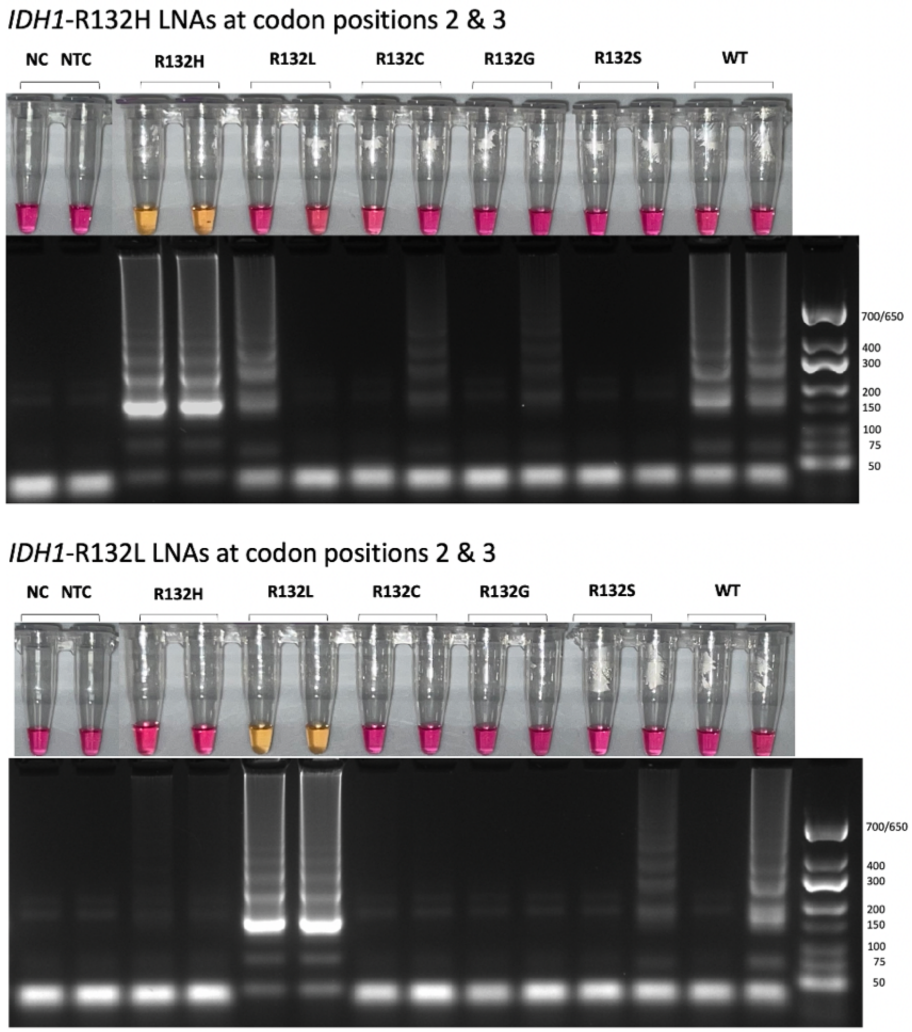


**Supplemental Figure 4. Two LNAs, one at the SNV and another at the following base, mediate specificity less robustly than other primers tested with *IDH1*-R132H.** In repetitions of this experiment, amplification was occasionally noted with non-target DNA (data not shown). Sample order is as follows: 1. NC (molecular grade $H_2O$), 2. NTC (*P. aeruginosa*), 3-4. *IDH1*-R132H DNA at 5.0X$10^4$ copies, 5-6. *IDH1*-R132L DNA at 5.0X$10^4$ copies, 7-8. *IDH1*-R132C DNA at 5.0X$10^4$ copies, 9-10. *IDH1*-R132G DNA at 5.0X$10^4$copies, 11-12. *IDH1*-R132S DNA at 5.0X$10^4$ copies, 13-14 *IDH1*-R132 wildtype DNA at 5.0X$10^4$ copies. Reactions were incubated at 68°C for approximately 35 minutes. These results are representative of multiple experiments. Similar results are found when assaying the other second position variant, *IDH1*-R132L, with LNAs at codon positions 2 and 3.

**Supplemental Table 1.** Pathology report of glioma tumor lysates utilized in figures 5A-5C and supplemental figures 5a-d. This report was provided to us prior to the release of the CNA5 by the World Health Organization (WHO). While samples 7 and 8 are listed as glioblastoma (GBM), new classifications place astrocytoma with IDH mutations in a separate category from GBM (11).

| Sample | Diagnosis | *IDH1* Pathology Report | LAMP Result |
|---|---|---|---|
| **1** | Glioblastoma multiforme | Negative | Negative |
| **2** | Glioblastoma multiforme (WHO grade IV astrocytoma) | Negative | Negative |
| **3** | Glioblastoma multiforme (WHO grade IV) | Negative | Negative |
| **4** | Glioblastoma multiforme (WHO grade IV) | Negative | Negative |
| **5** | Glioblastoma multiforme | Negative | Negative |
| **6** | Glioblastoma multiforme (WHO grade IV) | Negative | Negative |
| **7** | Glioblastoma | Positive in a subpopulation of the tumor | Positive |
| **8** | Glioblastoma | Positive in a subpopulation of the tumor | Positive |
| **9** | High-Grade Glioma | Positive | Positive |
| **10** | Anaplastic astrocytoma | Positive | Positive |

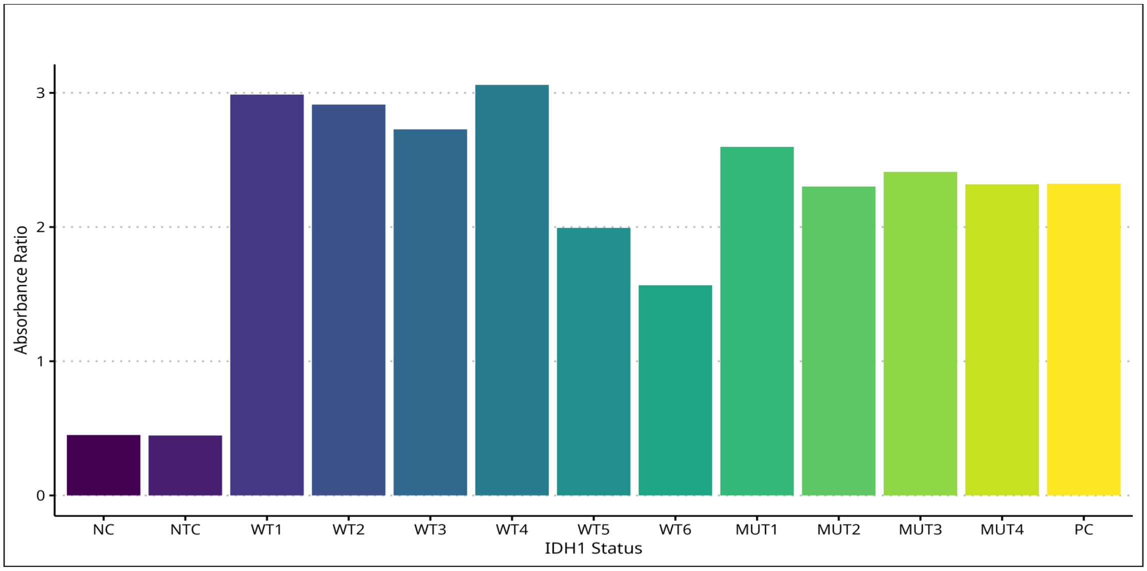

**Supplemental figure 5. Post-amplification absorbance ratios for glioma tumor samples using IDH1-R132 wildtype specific primers**. Colorimetric changes and sample order correspond with Figure 5A in the text body.

**Supplemental Table 2.** Additional statistical information for the analysis of figure 5D

*Pairwise Two-sample Wilcoxon Test of Absorbance Ratio by IDH1 Status*

| Group 1 | Group 2 | N1 | N2 | Statistic | *p* |
|---|---|---|---|---|---|
| Neg. Control | Wildtype | 8 | 24 | 45.00 | .155 |
| Neg. Control | Pos. Control | 8 | 4 | 0.00 | .024* |
| Neg. Control | Mutant | 8 | 16 | 0.00 | < .001*** |
| Wildtype | Pos. Control | 24 | 4 | 0.00 | .001*** |
| Wildtype | Mutant | 24 | 16 | 0.00 | < .001*** |
| Pos. Control | Mutant | 4 | 16 | 57.00 | .094 |

*Note.* The displayed p-values are the Bonferroni adjusted p-values.

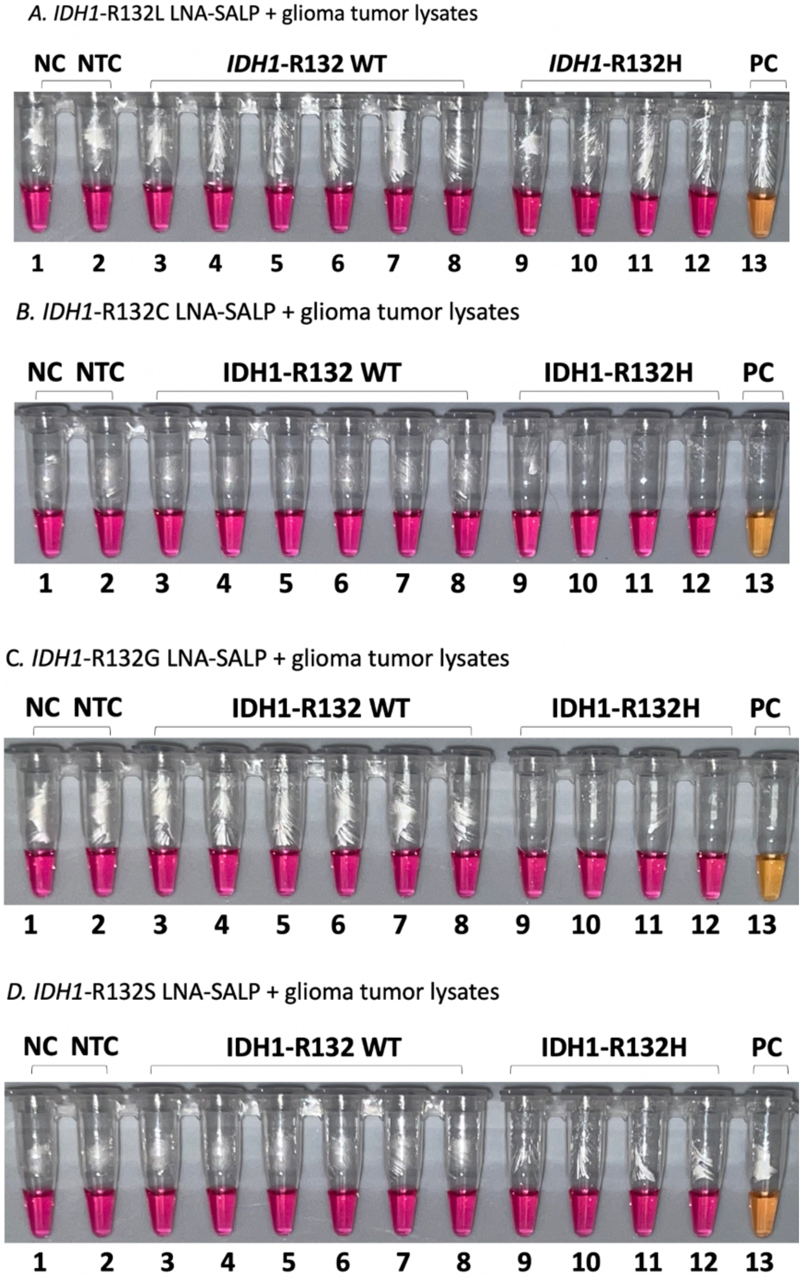


**Supplemental Figure 6A-6D. *IDH1*-R132H and *IDH1*-R132 wildtype glioma tumor samples show no visual evidence of amplification when combined with non-complementary LNA-SALPs.** Sample order is as follows: 1. NC, 2. NTC, 3-8 *IDH1*-R132 wildtype glioma tumor lysates, 9-12 *IDH1*-R132H mutant glioma tumor lysates, 14. *IDH1*-R132 synthetic DNA complementary to each respective primer set tested at 1.0X$10^6$ copies. Absorbance ratios for these samples can be found in supplemental figure 7. Reactions were incubated at 68$^o$C for approximately 35 minutes. These results are representative of multiple experiments.

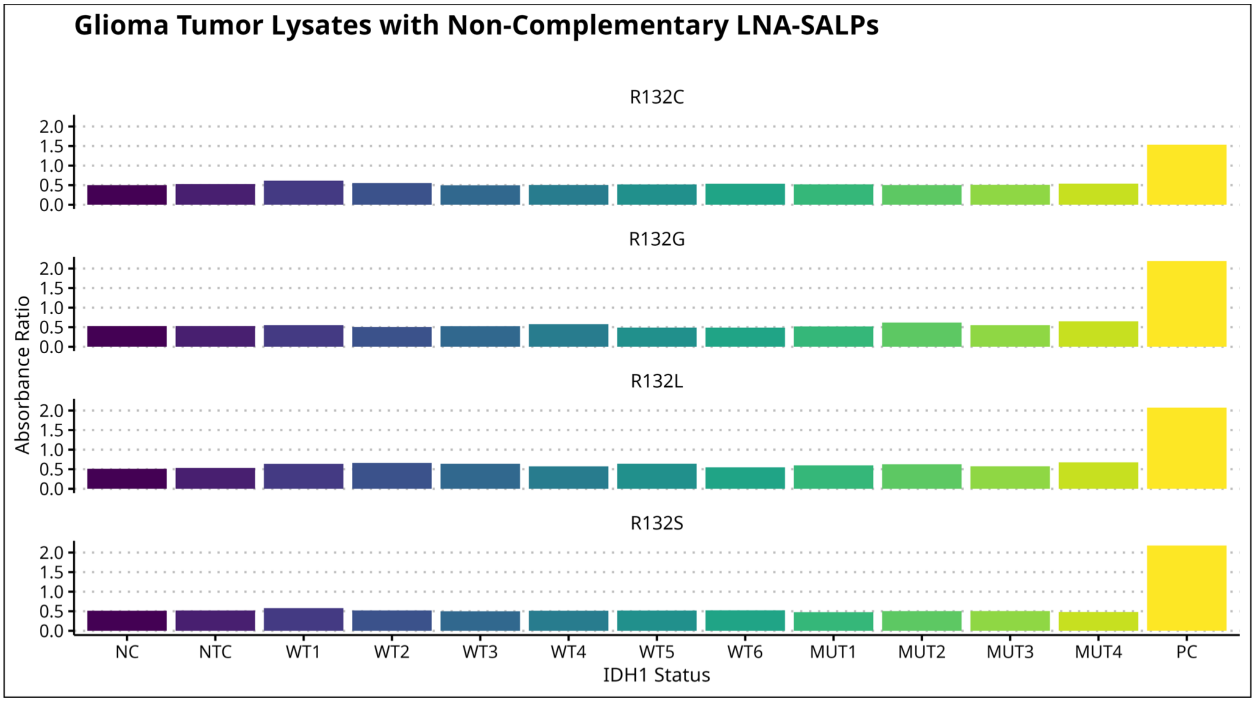


**Supplemental Figure 7. *IDH1*-R132H and *IDH1*-R132 wildtype glioma tumor samples show no evidence of amplification via absorbance ratio when combined with non-complementary LNA-SALPs.** Corresponding colorimetric changes, along with sample order and type, can be found in supplemental figure 6.
